# Measuring Outcomes in a Community Resilience Program: A New Metric for Evaluating Results at the Household Level

**DOI:** 10.1371/currents.dis.15b2d3cbce4e248309082ba1e67b95e1

**Published:** 2016-09-28

**Authors:** David P. Eisenman, Rachel M. Adams, Helene Rivard

**Affiliations:** David Geffen School of Medicine & Fielding School of Public Health, Center for Public Health and Disasters, University of California, Los Angeles, Los Angeles, California, USA; Department of Community Health Sciences, Fielding School of Public Health, University of California, Los Angeles, Los Angeles, California, USA

## Abstract

Community resilience programs require metrics for evaluation but none exist for measuring outcomes at the household and neighborhood level.

Objectives: We develop and describe a new index, the LACCDR index of community resilience, to examine how resilience varied across communities at baseline, prior to implementation of the Los Angeles County Community Disaster Resilience Project (LACCDR).

Methods: We surveyed 4700 adult residents in the sixteen LACCDR communities in English, Spanish and Korean. Each of the survey domains were selected a priori as outcome indicators aligned to the theoretical levers of community resilience. Survey questions were drawn and adapted from published studies and national surveys.

Results: Factor analysis demonstrated five separate factors composed from 18 items and explaining 46.7% of the variance. The factors were characterized as community engagement, emergency supplies, communication with neighbors, civic engagement, and collective efficacy. Baseline results for the 16 communities are provided.

Conclusions: We conclude that the LACCDR community resilience index can be used to measure resilience program outcomes at the neighborhood and household levels.

## Introduction

Community resilience— a community’s sustained ability to withstand and recover from adverse events—has progressed from theory to becoming the dominant framework guiding disaster preparedness and recovery planning and programming globally.^1^^,^ 2 ^,^ 3 ^,^ 4 U.S. federal policy emphasizes using a “whole community” approach through local collaboration between governmental and nongovernmental agencies and empowering and engaging communities to contribute to their own self-sufficiency, planning and collective community response.^5^^,^ 6

In the U.S. and across the globe, government and nongovernment agencies are implementing and testing community resilience programs that aim to empower communities to protect against, mitigate, respond to, and recover from threats and hazards. At the global level, the Rockefeller Foundation’s 100 Resilient Cities program aims to improve city-level resilience to shocks and disruption as varied as natural disasters, chronic unemployment, and food shortages.^7^ Nationally, America’s PrepareAthon, led by the Federal Emergency Management Agency, is a community-based program that aims to increase community engagement in resilience planning and improve participants’ knowledge of hazards and how to stay safe and mitigate damage.^8^ American Red Cross, too, is working on community resilience in many of its programs, some of which include community engagement as a component.^9^ Other local-level programs include the Neighborhood Empowerment Network and the Community and Regional Resilience Institute.^10^^,^ 11

The effectiveness of such programs remains largely unproven. Evaluating the outcomes of community-level resilience programs is critical and cannot proceed without meaningful metrics. To our knowledge, there are no published tools for measuring the outcomes of community-based resilience at the neighborhood or household level.^2^^,^ 3 ^,^ 4 ^,^ 10 ^,^ 11 The Conjoint Community Resilience Assessment Measurement was developed and tested in Israel. It assesses leadership, collective efficacy, preparedness, place attachment, social trust and social relationships in order to provide an assessment of communities’ resilience in Israel and is not intended as an outcome measure.^12^ The National Health Security Preparedness Index is a U.S. based tool that summarizes health security preparedness, but it tracks resilience of larger jurisdictions including states and metropolitan areas.^13^

In this paper, we present the LACCDR index of community resilience developed for the Los Angeles County Community Disaster Resilience Project (LACCDR). We describe how the index was constructed and then examine how resilience varies across communities in the LACCDR project at baseline, prior to implementation of the LACCDR program. The overall goal of this paper is to inform future evaluations of disaster resilience programs both in the U.S. and globally that aim to improve community resilience and measure outcomes at the neighborhood and household levels.

## Methods

The Los Angeles County Community Disaster Resilience project is a metropolitan public health agency led effort to enhance disaster resilience at the community level through the operationalization of four theoretical levers of resilience: education, engagement, self-sufficiency, and partnership.^3^ As defined by Chandra et al., education ensures ongoing information about preparedness, risks and resources before, during, and after a disaster. Engagement involves including community members and promoting participatory decision making in planning, response and recovery activities. Self-sufficiency refers to enabling and supporting individuals and communities to assume responsibility for their preparedness. Organizational partnership involves increasing and enhancing the linkages and collaborations between government and non-governmental organizations (NGOs) and between NGOs in the community. In brief, sixteen communities were randomly assigned to receive an experimental community resilience program or a comparison program. Public health nurses trained community coalitions to utilize a resilience toolkit in the experimental arm. The comparison communities received training in traditional disaster preparedness topics, such as household supplies and emergency communication plans. The project’s goals, development and methods are described in previous publications.^14^^,^ 15 ^,^ 16 ^,^ 17

We needed new strategies and metrics to measure project outcomes in LACCDR. We used the Public Health Response to Emergent Threats Survey (PHRETS), a population-level survey periodically fielded in Los Angeles County to guide disaster planning, to be the vehicle for evaluating LACCDR outcomes at the household and neighborhood level. The PHRETS baseline instrument was rewritten to measure several domains related to community resilience. Each of these survey domains were selected a priori as outcome indicators aligned to the theoretical levers of community resilience of Chandra and colleagues.^3^ These domains included: household preparedness for disaster; participation in community resilience building activities; self-efficacy for helping in a disaster; perceived collective efficacy of the community in a disaster; perceived benefits of disaster planning with neighbors; social networks available in a disaster; and, civic engagement. Survey questions were drawn from published studies and national surveys, and adapted to fit this project’s evaluation.^18^^,^ 19 ^,^ 20 ^,^ 21

We collected baseline survey data from adult residents (≥18) of the sixteen LACCDR communities between June 3 and August 7, 2013. The address-based sample was selected to be representative of the sex, age and race/ethnicity distribution of the 2010 census tracts in each of the communities. The survey was administered by landline and mobile telephone in English, Spanish and Korean. The study protocol was reviewed and approved by the institutional review board of the Los Angeles County Department of Public Health.

**Data Analysis**

Prior to analysis, the data were weighted using raking ratio estimation. 2010 census blocks were used to determine the population benchmarks for age, sex and race/ethnicity. We imputed missing values for the weighting variables using the modal responses for each community prior to raking. Raked weights were trimmed at the 2nd and 98th percentiles to decrease variance.

Survey questions were scaled so that the responses ranged from zero to one. This scaling method provided an equal weight for each question in the analysis. Higher values represented agreement with community resilience questions, so that binary questions were scored 0 for “no” and 1 for “yes.” Likert-scale questions were scaled so that the responses ranged evenly from 0 for responses that reflected strongest disagreement, to 1 for responses that reflected strongest agreement. We reverse coded all negatively keyed questions.

Principal component analysis extraction was used to help determine how many factors existed across the 18 community resilience variables of interest. We determined the number of factors using the Scree test and examining how many eigenvalues were greater than 1. Factor analysis with Varimax rotation followed. We retained questions and separated factors using a 0.40 factor loading cutoff. A Cronbach alpha value was generated for each of the factors in order to assess the reliability of the community resilience domains and the overall index.

Summary variables were generated for each of the identified factors by calculating the averages across each of its measures and multiplying this value by 100% for ease in interpretability. The calculations followed the model of the National Health Security Preparedness Index.^13^ An example of this calculation follows:

Factor 5 was comprised of three measures:

x_1_="Planning with my neighbors will help my household after a major disaster."

x_2_="I am confident I can be of help to my neighbors or community in the event of a disaster, such as an earthquake."

x_3_="People in my neighborhood know how to work together to prepare and respond to a disaster."

Where for each observation i,

x_1i_, x_2i_, x_3i_= {0="Strongly Disagree"; 0.333="Disagree"; 0.667="Agree"; 1="Strongly Agree"}

The summary factor score was calculated by taking the average of these two measures, and multiplying the result by 100:

Factor­_5i_=[(x_1i_+x_2i_+x_3i_)/3]*100%

The result was a number between 0 and 100 for each observation i.

Summary variables for each of the factors were examined across each of the sixteen communities. An overall community resilience index was then calculated by taking the average of the composite scores for each of the factors.

If an observation was missing a value or had a response of “Don’t know” or “Refused,” then a score was not calculated for that observation. This method of handling missing responses, listwise deletion, has been used in another study similar to ours^12^ and is the default method for treating missing data in SAS version 9.4 (SAS Institute, Inc., Cary, North Carolina). We also conducted a post-hoc missing data analysis to evaluate whether there were differences in the demographic characteristics of missing and non-missing observations.

## Results

Data from 4700 respondents were included in this analysis (35% response rate). Despite recruiting respondents from a representative address-based sample, the low response rate led to underrepresentation of males, younger respondents (ages 18-29 and 30-44), Hispanics, and Asians. Weighting of sample respondents according to the age, sex and race/ethnicity distribution of the 2010 census tracts helped enhanced the representativeness of the sample. Table 1 presents the socio-demographic characteristics of this sample population after weighting. The majority of respondents were female (52%), between 30 and 44 years old (31%), white (40%), possessed a college degree or higher (34%), earned a household income between $10,000 and $30,000, were married or living with partner (57%), spoke mostly English in the home (57%), and reported no health limitations (79%).

**Table 1:** Table 1. Sample characteristics of respondents after weighting, Public Health Response to Emergent Threats Survey 2013 (N=4700)

Variables		N (%)
Sex	Male	2274 (48%)
	Female	2418 (52%)
Age	18-29	1026 (22%)
	30-44	1445 (31%)
	45-59	1260 (27%)
	60+	941 (20%)
Race/Ethnicity	White	1829 (40%)
	African American	440 (10%)
	Asian	471 (10%)
	Hispanic	1537 (34%)
	Other	239 (5%)
Income	<$10,000	444 (11%)
	$10,000-29,999	1157 (29%)
	$30,000-49,999	842 (21%)
	$50,000-99,999	928 (23%)
	>$100,000	652 (16%)
Education	Some High School or Less	691 (15%)
	High School Graduate/GED	1164 (25%)
	Associate Degree/Trade School/Some College	1243 (26%)
	College Degree or Higher	1602 (34%)
Marital Status	Married/Living with a partner	2610 (57%)
	Divorced/Separated/Widowed	676 (15%)
	Never married	1288 (28%)
Language Most Used in Home	English	3068 (66%)
	Spanish	1262 (27%)
	Other	310 (7%)
Any Health Limitations	Yes	978 (21%)

Factor analysis demonstrated five separate factors, which together accounted for 46.71% of the explained variance (Table 2). Based on the items meeting the 0.4 factor loading threshold, the factors were characterized as (1) community engagement, (2) emergency supplies, (3) communication with neighbors, (4) civic engagement, and (5) collective efficacy.

Community engagement (Cronbach α=0.68) included measures of having “attended a community meeting where preparing for emergencies or disaster was discussed,” “worked or volunteered to help your neighborhood or community prepare for or respond to a disaster or emergency,” “worked or volunteered with a group of organization that focused on community safety, such as Neighborhood Watch,” “attended a training to help others in your community in a disaster or emergency, like first aid or CPR,” “attended a training in psychological first aid,” and “looked for information about getting prepared for a disaster, for example on the internet or by calling the Department of Public Health.”

Emergency supplies (Cronbach α=0.54) described having the following household resources: a 3-day supply of water per person, a 3-day supply of non-perishable foods per person, a 7-day supply of prescription medication per person, a household plan to reunite, and additional emergency supplies useful in a disaster.

Communication with neighbors (Cronbach α=0.38) was characterized as “talk[ing] with a neighbor about preparing for an emergency or disaster” and “hear[ing] or see[ing] any messages telling you to plan together with your neighbors for an emergency or disaster.”

Civic engagement (Cronbach α=0.67) included belonging to a “community organization (e.g. school, church or other faith, community or volunteer organization) that you can depend upon in a disaster” and participating in “meetings or activities with voluntary organizations or associations like school groups, churches or temples, community centers, ethnic associations or other social or civic organizations” in the past 12 months.

Perceived collective efficacy (Cronbach α=0.34) measured agreement with the following statements: “planning with my neighbors now won’t help my household after an earthquake or other major disaster,” “I am confident I can be of help to my neighbors or community in the event of a disaster, such as an earthquake,” and “people in my neighborhood know how to work together to prepare and respond to a disaster.”

**Table 2:** Table 2. Results from factor analysis of community resilience measures, Public Health Response to Emergent Threats Survey 2013 (N=4700)

	Factor 1	Factor 2	Factor 3	Factor 4	Factor 5
Survey Measure	Factor Loadings				
Have you attended a community meeting where preparing for emergencies or disasters was discussed?	0.48	0.08	0.36	0.22	0.08
Have you worked or volunteered to help your neighborhood or community prepare for or respond to a disaster or emergency?	0.55	0.12	0.38	0.10	0.06
Have you worked or volunteered with a group or organization that focuses on community safety, such as a Neighborhood Watch?	0.45	0.05	0.33	0.18	0.17
Have you attended a training to help others in your community in a disaster or emergency, like first aid or CPR?	0.73	0.04	-0.05	0.02	0.13
Have you attended a training in psychological first aid?	0.71	0.03	-0.02	0.02	0.00
Have you looked for information about getting prepared for a disaster, for example on the internet or by calling the Department of Public Health?	0.46	0.30	0.02	0.10	0.03
A 3-day supply of water is one gallon of water per person per day. Does your household have a 3-day supply of water for each person who lives there?	0.07	0.65	0.14	-0.05	0.00
Non-perishable foods do not need refrigeration or cooking, such as canned or packaged meat, soups, fruits and vegetables. Does your household have a 3-day supply of non-perishable food for each person who lives there?	-0.01	0.69	0.02	0.04	0.12
Does your household have a 7-day supply of prescription medications for each person who takes prescribed medications?	-0.01	0.48	-0.24	0.10	0.06
Does your household have a plan for how you will find each other or reunite if you are separated in a disaster?	0.25	0.42	0.14	0.01	0.15
Have you bought additional emergency supplies of food, water, first aid supplies or other tools or items useful in a disaster?	0.17	0.61	0.20	0.09	0.03
Have you talked with a neighbor about preparing for an emergency or disaster?	0.19	0.26	0.50	0.10	0.29
Did you hear or see any messages telling you to plan together with your neighbors for an emergency or disaster?	0.05	0.04	0.74	-0.01	-0.05
Do you belong to a community organization (e.g., school, church or other faith community, or volunteer organization) that you can depend upon in a disaster?	0.10	0.06	0.11	0.85	0.07
In the past 12 months, how often did you participate in meetings or activities with voluntary organizations or associations like school groups, churches or temples, community centers, ethnic associations or other social or civic organizations?	0.17	0.07	-0.01	0.84	0.04
Planning with my neighbors now won't help my household after an earthquake or other major disaster.	0.06	-0.02	-0.33	0.10	0.64
I am confident I can be of help to my neighbors or community in the event of a disaster, such as an earthquake.	0.22	0.13	0.07	-0.03	0.60
People in my neighborhood know how to work together to prepare and respond to a disaster.	-0.08	0.17	0.30	0.09	0.65
Eigenvalues	3.86	1.52	1.34	1.12	1.04
Percent of variance explained	20.31	8.00	7.07	5.88	5.45
Cronbach's Alpha coefficient	0.68	0.54	0.38	0.68	0.34

Table 3 presents the results of the resilience scores across each of the sixteen communities. For the total community resilience index, Acton/Agua Dulce (47.5%) and Culver City (43.6%) had the highest score whereas San Gabriel (34.2%) and Hollywood (36.7%) had the lowest. While the community engagement factor was low across all communities (16.3% total), Watts (22.6%), Acton/Agua Dulce (18.5%) and Compton (18.4%) possessed the three highest values. Emergency supplies scores were generally higher across all communities (58.3% total average), with Acton/Agua Dulce having a score above 70%. For the communication with neighbors factor (21.5% total score), there was greater variation across the different communities. Watts (32.8%) scored almost three times higher than San Gabriel (12.7%) in this domain. Civic engagement was more similar across neighborhoods (38.6% total), with Acton/Agua Dulce (44.3%), Compton (41.4%), Culver City (40.3%), La Crescenta (45.4), Palms (43.5%), and Watts (41.9%) possessing above average values. Collective efficacy was the community resilience domain with the highest overall score (63.2% total). Only Hawaiian Gardens (57.9%), Huntington Park (58.2%) and Watts (57.5%) had values below 60%.

**Table 3:** Table 3. Community resilience scores for each neighborhood, Public Health Response to Emergent Threats Survey 2013 (N=4700)

	Factors (% Score)					
	Community Engagement	Emergency Supplies	Communication with Neighbors	Civic Engagement	Collective Efficacy	Total Score
Overall Sample	16.3	58.3	21.5	38.6	63.2	40.1
Acton/Agua Dulce	18.5	71.3	22.6	44.3	74.5	47.5
Compton	18.4	57.2	23.3	41.4	60.0	39.5
Culver City	17.2	61.0	22.6	40.3	68.1	43.6
Gardena	16.8	56.2	20.1	37.3	61.8	38.7
Hawaiian Gardens	14.3	55.1	27.1	33.5	57.9	38.2
Hollywood	11.8	57.8	17.1	30.9	63.1	36.7
Huntington Park	14.8	56.6	31.4	37.3	58.2	40.2
La Crescenta	16.0	62.8	18.8	45.4	68.0	43.0
Palms	17.0	51.0	19.5	43.5	61.6	39.5
Pico Union	15.9	62.1	18.4	37.1	63.0	39.8
Pomona	14.6	53.8	21.3	38.4	62.8	38.7
Quartz Hill	16.8	58.5	15.0	38.6	67.8	40.4
San Fernando	16.2	58.2	22.2	35.7	63.0	39.0
San Gabriel	15.0	57.1	12.7	31.1	60.6	34.2
Watts	22.6	55.7	32.8	41.9	57.5	42.4
Wilmington	14.8	59.2	23.8	37.8	60.1	39.9

## Discussion

We built and tested a LACCDR community resilience index that allows us to quantify and understand baseline resilience at the neighborhood level. This index will allow us measure the impact of the LACCDR initiative and can also guide other public health agencies in their evaluation efforts.

Exploratory factor analysis demonstrated that community resilience can be divided into five distinct domains. These factors overlap with the Chandra and colleague levers used to guide the development of the survey, supporting the structure of their framework and grounding our community resilience in a theoretical framework.^3^ The community engagement factor aligns to the Chandra’s engagement lever and education lever.^16^ The emergency supplies factor aligns with the self-sufficiency lever as they both focus on personal preparedness. The communication with neighbors and collective efficacy factors map to the partnerships lever, which calls for engaging social networks (and local organizations) to develop and disseminate preparedness information and supplies.^3^

The five community resilience domains in the LACCDR index have similarities and differences with measures used in other community disaster resilience tools. Leykin et al.’s Conjoint Community Resilience Assessment Measure, which was developed to examine resilience among Israelis, is based on leadership, collective efficacy, preparedness, place attachment and social trust.^12^ Like our results, collective efficacy describes perceptions of a community’s ability to work together by including such items as “I can depend on people in my town to come to my assistance in a crisis” and “I believe in the ability of my community to overcome an emergency situation.”^12^ Social trust and preparedness also overlap with some of our measures in collective efficacy and emergency supplies. Place attachment, on the other hand, introduces a resilience concept that describes one’s psychological connection with the environment. While important to community resilience, place attachment questions were not included in our survey as they were less amenable to change through the LACCDR public health intervention.

Pfefferbaum et al.’s Communities Advancing Resilience Toolkit (CART) survey, which was not published at the launch of LACCDR, was developed as a community assessment tool that provides a “snapshot of strengths and challenges”, not as an outcome measure that can be fielded in a pre-post manner.^22^ It categorizes community resilience into four domains: connection and caring, resources, transformative potential, and disaster management.^22^^,^ 23 As CART focuses on the respondent’s perceptions of his or her community’s disaster resilience to disaster, it has measures overlapping with our collective efficacy domain, but does not capture participatory behaviors such helping the neighborhood prepare for disaster, attending relevant trainings, and talking with neighbors about self-sufficiency.

The LACCDR index has several benefits as an outcome measure. First, by including a variety of diverse measures, it captures the multi-dimensionality of community resilience. While some of the factors did not have the high measures of internal consistency, together they contributed to a more reliable community resilience index (Cronbach α=0.61). Second, we outline straightforward calculations that can be easily replicated. This will not only help us compare our baseline results to future survey data, but other community resilience programs can use it as a guide for evaluating their outcomes. Finally, we developed an index that is flexible. The overall resilience index is comprised of separate factors that can be assigned separate weights. Researchers may want adjust weights to accentuate factors associated with evidence-based outcomes or to evaluate programs that place greater emphasis on certain domains over others.

The LACCDR community resilience index used a targeted population and weighted sample of respondents, so that it is possible to get a good representation of the level of community resilience in each neighborhood. Understanding the different characteristics of each community lends itself to interpreting baseline resilience trends. For instance, Acton and Agua Dulce, geographically isolated towns in the high desert region of the county, possessed the highest scores for community supplies and collective efficacy. One possible explanation for these findings is that residents may be especially aware of their vulnerability in a disaster, which is supported by our experience in these communities. Stockpiling household resources, such as water, can be particularly useful if people are stranded in a harsh climate during an emergency. Furthermore, the community’s isolation from other towns may foster a culture of preparedness among neighbors.

In addition to geography, socio-economic status may also be contributing to the high community supplies and collective efficacy scores in Acton and Agua Dulce. These communities possesses the highest median household income of all the participating communities.^17^ Past emergency preparedness research supports the positive relationship between income level and possessing household supplies and emergency plans.^24^^,^ 25 ^,^ 26 ^,^ 27 Culver City and La Crescenta, two other high-income areas, similarly display this trend. With the three wealthiest study populations also possessing the highest total community resilience scores, it appears as though the baseline results may be influenced by socio-economic status.

Watts possessed the highest scores for community engagement and communication with neighbors and also had a high score for civic engagement. Ever since the 1965 Watts Rebellion, when riots broke out over racial tensions surrounding police brutality, community members have been actively involved in civic organizations, including community empowerment programs and churches.^28^^,^ 29 The existing infrastructure of faith- and community-based organizations may provide opportunities to build disaster resilience within this community.

San Gabriel was the neighborhood with the lowest total resilience score. This community is home to many Asian immigrants speaking a variety of languages. In this survey sample, 39% of San Gabriel residents indicated that they did not speak English or Spanish in the home. Language barriers may therefore be contributing to lower community engagement and communication with neighbors.^30^

**Limitations**

Some limitations to this analysis should be acknowledged. The study sample was selected from sixteen Los Angeles County communities and may not be representative of the county as a whole. The low response rate may also reduced the representativeness of the target population due to systematic non-response from the survey sample. However, we attempted to address this bias by weighting the respondents according to the age, sex and race/ethnicity distribution of the 2010 census tracts in each of the communities. Applying population weights has been show to enhance the representativeness of the sample and reduce threats to validity caused by low response rates.^31^^,^ 32 ^,^ 33 In addition, missing response analysis revealed demographic differences among respondents with missing values, especially for the items in the collective efficacy factor. Missing responses occurred more frequently among those who are female, 60 years of age or older, white, possess a college degree or higher, and predominantly speak English at home. Underrepresentation of these groups may have biased the results.

## Conclusion

This analysis illustrates how to assess disaster resilience at the community level. Using the LACCDR community resilience index, we describe baseline neighborhood measures that can be used to track changes over time. These factor scores supplement qualitative evaluations that have been conducted throughout the course of the intervention. Once follow-up data is collected, we can use this index to investigate the impact of the Los Angeles County Community Disaster Resilience initiative.

## Corresponding Author

David P Eisenman, MD MSHS, Professor of Medicine and Public Health, David Geffen School of Medicine at UCLA and UCLA Fielding School of Public Health, Director - UCLA Center for Public Health and Disasters. deisenman@mednet.ucla.edu

## Data Availability Statement

The data used for this analysis can be accessed via https://dx.doi.org/10.6084/m9.figshare.3202120.v1

## Competing Interest Statement

The authors have declared that no competing interests exist.
